# Distorted gaze direction input to attentional priority map in spatial neglect

**DOI:** 10.1016/j.neuropsychologia.2019.05.017

**Published:** 2019-08

**Authors:** Daniela Balslev, Bartholomäus Odoj

**Affiliations:** a School of Psychology and Neuroscience, University of St Andrews, St Andrews, KY169JP, UK; b Center of Neurology, Division of Neuropsychology, Hertie Institute for Clinical Brain Research, University of Tuebingen, Tuebingen, 72076, Germany; c Department of Psychology, University of Copenhagen, Copenhagen, DK, 1353, Denmark

**Keywords:** Attention, Stroke, Spatial neglect, Coordinate transformation

## Abstract

A contribution of the gaze signals to the attention imbalance in spatial neglect is presumed. Direct evidence however, is still lacking. Theoretical models for spatial attention posit an internal representation of locations that are selected in the competition for neural processing resources – an attentional priority map. Following up on our recent research showing an imbalance in the allocation of attention after an oculoproprioceptive perturbation in healthy volunteers, we investigated here whether the lesion in spatial neglect distorts the gaze direction input to this representation.

Information about one's own direction of gaze is critical for the coordinate transformation between retinotopic and hand proprioceptive locations. To assess the gaze direction input to the attentional priority map, patients with left spatial neglect performed a cross-modal attention task in their normal, right hemispace. They discriminated visual targets whose location was cued by the patient's right index finger hidden from view. The locus of attention in response to the cue was defined as the location with the largest decrease in reaction time for visual discrimination in the presence vs. absence of the cue. In two control groups consisting of healthy elderly and patients with a right hemisphere lesion without neglect, the loci of attention were at the exact location of the cues. In contrast, neglect patients allocated attention at 0.5⁰-2⁰ rightward of the finger for all tested locations. A control task using reaching to visual targets in the absence of visual hand feedback ruled out a general error in visual localization. These findings demonstrate that in spatial neglect the gaze direction input to the attentional priority map is distorted. This observation supports the emerging view that attention and gaze are coupled and suggests that interventions that target gaze signals could alleviate spatial neglect.

## Highlights

• The mechanisms of left inattention in spatial neglect are incompletely understood.
• Attention loci in visual space are displaced to the right of somatosensory cues.
• This indicates a distorted gaze direction input to the attentional priority map.
• Distorted gaze direction input could lead to left-right attention imbalance.

## Graphical abstract

**Figure:**
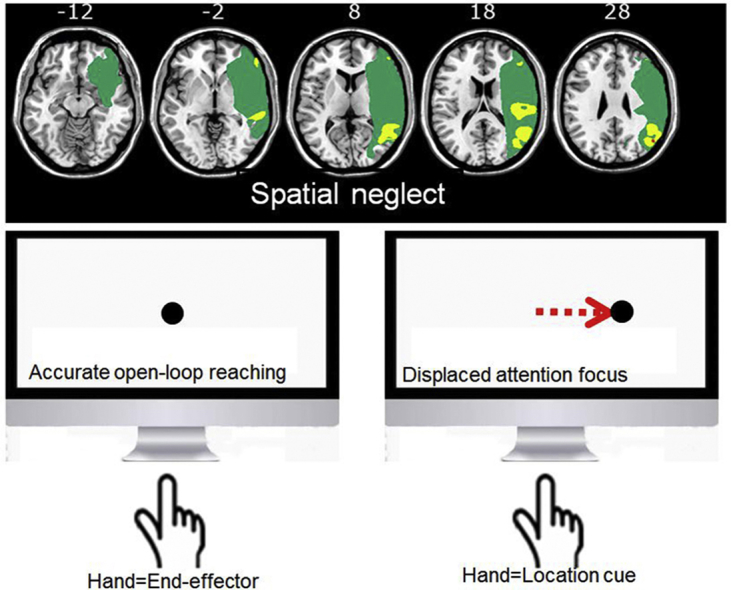

## 1 Introduction

Spatial neglect is a common and disabling outcome after a stroke (Buxbaum et al., 2004; Ferro et al., 1999). It is defined as the inability to report, respond or orient to stimuli presented in contralesional space despite adequate sensory and motor abilities (Heilman et al., 1985). The most persistent clinical sign is a spatial imbalance in the allocation of attention (Farne et al., 2004; Rengachary et al., 2011). For instance, after a left hemisphere lesion, the further to the right the visual stimuli, the faster and the more accurate their detection (Smania et al., 1998). This left-right imbalance has more recently been explained by a disruption of an attentional priority map (Ptak and Fellrath, 2013). The priority map is an internal representation that encodes the location of salient or goal-relevant objects regardless of their features (Bisley and Goldberg, 2010; Fecteau and Munoz, 2006; Shomstein and Gottlieb, 2016). This representation acts on topographically organized, lower-level sensory areas to prioritize input arriving from those locations (Luck et al., 2000; Ruff et al., 2006; Yantis et al., 2002). Within this theoretical framework, the attention imbalance in spatial neglect results from an under-representation of the contralesional locations on a priority map that guides the allocation of attention. There are a number of hypotheses why this representation is inaccurate and sensory input from contralesional space disregarded.

Firstly, the unilateral lesion could irreversibly damage neurons that encode contralesional locations on the attentional priority map, effectively reducing the space where attention can be allocated (Pouget and Driver, 2000; Vuilleumier et al., 2007).

Secondly, the attentional priority map could be displaced ipsilesionally following an ipsilesional displacement of the perceived midline of one's own body. The mechanism of the shift in perceived body midline is presumed to be an error in the coordinate transformations that convert visual, vestibular and neck proprioceptive input into internal representations of space (Jeannerod and Biguer, 1987; Karnath, 1997; Vallar, 1997). A lateral displacement of the attentional priority map is suggested by the patients' Gaussian distribution of spontaneous eye movements in darkness (Fruhmann-Berger and Karnath, 2005) or by the deviation of the perceived midsagittal plane in darkness (Karnath, 1994). This evidence is however, indirect. Although changes in the allocation of attention can accompany deviations in the perception of midsagittal plane (Martín-Arévalo et al., 2016; Rossetti et al., 1998), this is not always the case (Chokron, 2003; Rorden et al., 2001).

Thirdly, the map could fail to encode the location of some attention cues. To allow cross-modal interactions in spatial attention or to stabilise the attentional priority map across eye movements, a retinotopically organised priority map (Golomb et al., 2008) must be combined with information about the direction of gaze. There are two main signals that convey the rotation of the eyes in the orbits. One is the corollary discharge, which is a copy of the oculomotor command, the other is proprioceptive feedback from the extraocular muscles. It has been proposed that the lesion interferes with the ability to incorporate an eye movement signal, presumably the corollary discharge (Duhamel et al., 1992), into the attentional priority map (Pierce and Saj, 2018; Pisella and Mattingley, 2004). A failure to update spatial representations after eye movements would cause a deficit in retaining visual locations across saccades. Visual localisation errors after eye movements have indeed been observed in patients with spatial neglect (Duhamel et al., 1992; Heide and Kömpf, 1998; Husain et al., 2001). Under the assumption that visual localisation and visuospatial attention rely on the same gaze direction signals, one would expect the same errors in the allocation of attention. This assumption however, is challenged by our recent research.

We have observed that repetitive transcranial magnetic stimulation (rTMS) over an extraocular muscle proprioceptive area in the somatosensory cortex (Balslev and Miall, 2008) cause healthy volunteers to mislocalize visual targets relative to the hand when the hand serves as a location cue in a cross-modal attention task. In contrast, the same visual targets are correctly located when the hand serves as an effector for reaching, in the absence of visual hand feedback (Odoj and Balslev, 2016). We concluded that visuospatial attention and visual localisation for reaching rely on different combinations of eye position signals, and that oculoproprioception is weighted more in the eye position input to the attentional priority map. An error in the oculoproprioceptive signal after a lesion of the somatosensory cortex (Balslev et al., 2012) or after somatosensory cortex rTMS (Balslev and Miall, 2008) results in a reduced ability to locate visual stimuli relative to the body after passive movement of the eyeball. A more precise measure of the distortion in the perceived gaze direction after rTMS is provided by the amplitude of saccades from visual fixation points to auditory targets in darkness (Odoj and Balslev, 2013). The distortion in the eye position signal measured in this way matches in direction and extent the displacement of the attention loci in the visual space relative to finger position cues (Odoj and Balslev, 2016). Taken together, this previous work suggests a connection between the oculoproprioceptive input from the somatosensory cortex and the allocation of attention. We hypothesized therefore that the left-right attention gradient in patients with spatial neglect might be caused by distorted eye position input to the attentional priority map.

We investigated this hypothesis here. To assess the ability to allocate attention independently from the ability to locate visual objects, patients with spatial neglect were tested on a cross-modal attention task and a reaching task in the absence of visual hand feedback (open-loop). Importantly, both tasks require coordinate transformation between the visual and finger locations, a process that relies on an internal estimate of the direction of one's own gaze. Both tasks had been tested in healthy participants (Odoj and Balslev, 2016). In the cross-modal task patients discriminated visual targets whose location was cued by the location of their own right index finger hidden from view. Targets for visual discrimination were presented at that exact location, as well as at −3⁰, −2⁰, −1⁰, 1⁰, 2⁰ and 3⁰ from the finger position cue. The locus of attention was defined as the location with the largest decrease in reaction time for visual discrimination in the presence vs. the absence of a cue. Because perception efficiency was measured by the reaction time alone, the method requires that the accuracy for visual discrimination is at ceiling. All stimuli were therefore presented in the ipsilesional hemispace, where the patients' accuracy for visual discrimination did not differ significantly from that of the control groups. The two control groups consisted of healthy elderly and patients with a right hemisphere lesion without neglect. A distortion of the gaze direction input to the attentional priority map would cause a displacement of attention loci relative to attention cues for all tested locations. In contrast, a reduction of the map where the contralesional space is missing would not interfere with the allocation of attention in the ipsilesional hemifield, which is presumed to be normal. Likewise, because the locus of attention was defined by the difference in reaction time between a condition with an attention cue and a condition without an attention cue, an asymmetry in baseline, steady-state attention would impact equally on both conditions, leaving the difference between them unaffected. All experiments were performed at fixation, which was verified using an EyeLink II eye tracker. Therefore, a failure to retain visual location after eye movements is unlikely to explain current findings.

## 2 Materials and methods

### 2.1 Participants

Four neglect patients (one female) with a mean age of 63 years (range: 46–73) with brain lesions after a right hemisphere stroke participated in the study. Spatial neglect was diagnosed using Bells and Letter cancellation tasks (Albert, 1973; Gauthier et al., 1989). We calculated the center of mass for the detected targets (“center of cancellation”, CoC). A threshold of mean CoC = 0.083 was used as a threshold for left-sided neglect (Rorden and Karnath, 2010).

**Fig. 1:**
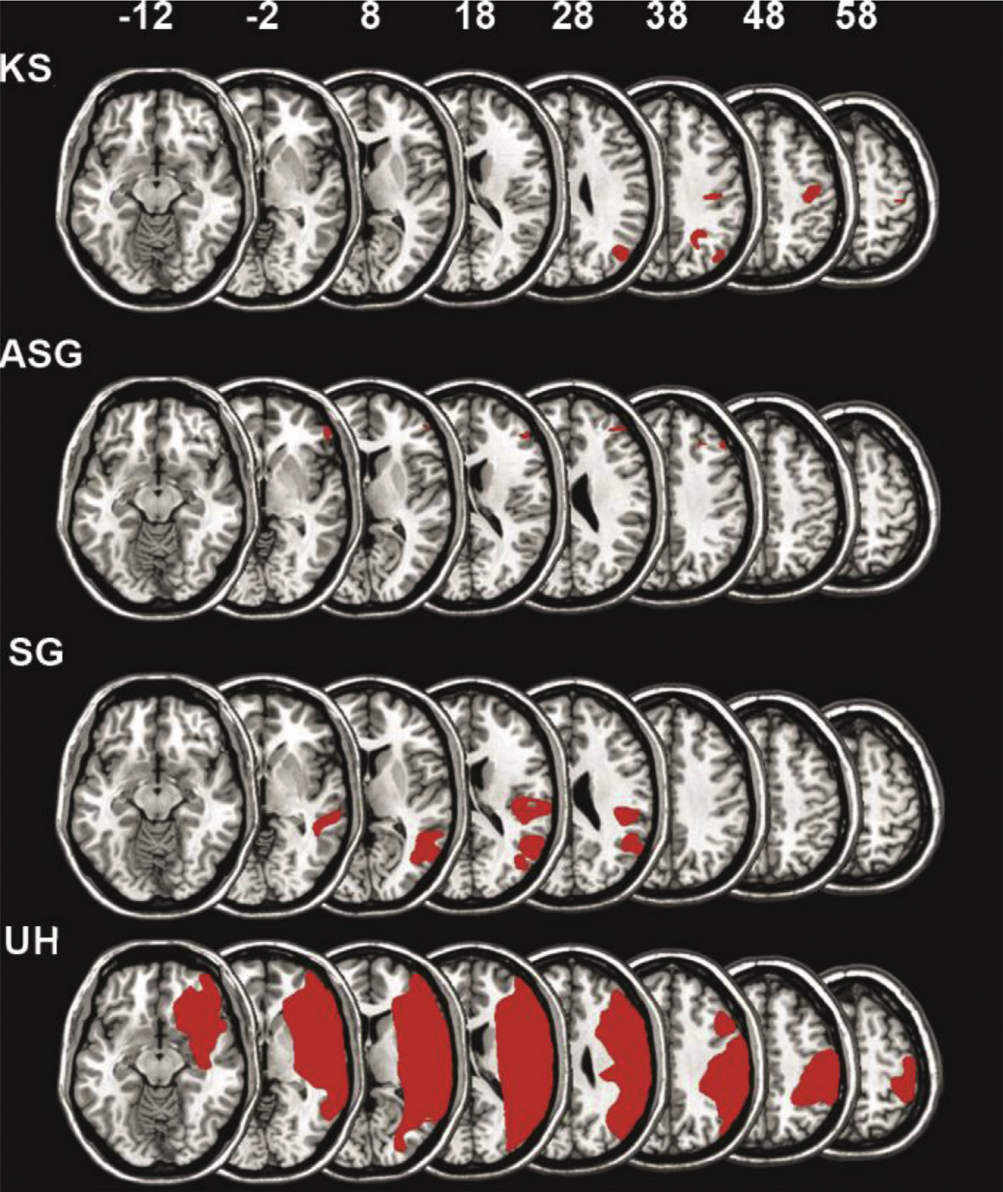
**Individual lesions of the patients with spatial neglect.** The colour maps indicating the lesion are superposed on a single-subject T1 template coregistered with the MNI152 template (International Consortium for Brain Mapping). The figure shows the vertical, *z* coordinate for each transversal slice. The left side of the brain is shown to the left. The demographics of the individual patients are listed in Table 1. (For interpretation of the references to colour in this figure legend, the reader is referred to the Web version of this article.)

**Fig. 2:**
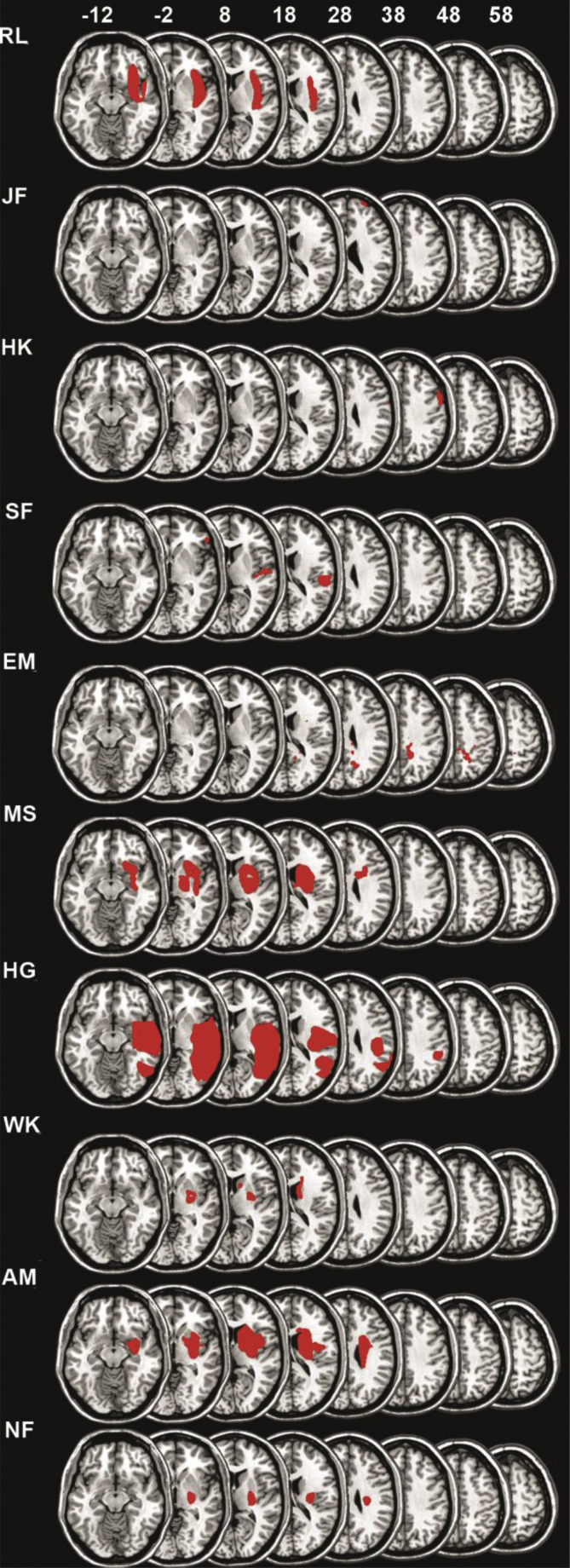
**Individual lesions of the patients in the control group.** Conventions like in Fig. 1.

We tested two control groups. The patient control group consisted of ten age-matched patients with lesions in the right hemisphere following a stroke, but no spatial neglect (mean age: 61.75 years; range: 43–81; independent samples t-test neglect vs. patient control group, p=.482). Table 1 provides demographic information and Fig. 1, Fig. 2 show the lesion of the individual patients with and without spatial neglect, respectively. There was no statistically significant difference between groups with respect to the time after the stroke and lesion size (Mann-Whitney U test, p=0.16 and p=0.64, respectively). The healthy control group consisted of ten healthy, age-matched participants (mean: 65 years; range: 62–74; independent samples t-test neglect vs. elderly control group, p=.285). All participants were right-handed.

**Table 1:** Patient demographics.

NEG	Post- stroke	Bells	Letter	Lesion Area	Lesion Size (cm^3^)

KS	1	0.376	0.492	rP, rO, rM	6.7
ASG	68	0.121	0.108	rF	2.1
SG	1	0.33	0.317	rT, rO, BG	19.0
UH	1	0.714	0.704	rP, rM, rF	233.5

PCG	Post- stroke	Bells	Letter	Lesion Area	Lesion Size (cm^3^)

RL	7	0.003	0	rP	31.57
JF	6	0.008	0.031	rF	0.2
HK	19	0.032	-0.01	rM	1.57
SF	18	0.059	0.065	rM, rT-P, rBG	6.3
EM	5	0	0	rM	4.4
MS	4	0.083	0	rBG	35.6
HG	17	0.008	0.011	rM	125.8
WK	10	0.061	-0.004	rM	5.4
AM	11	0.011	0	rBG	41.1
NF	15	0.005	0	rBG	6

**Note:** NEG-neglect patients, PCG – patient control group, “post-stroke” indicates the time of testing after the stroke, in months “Bells” and “Letter” indicate the individual CoC for the Bells and the Letter Cancellation tasks, respectively. “Lesion” describes lesion location for each patient: rBG-right basal ganglia, rF- right frontal, rM-right medial, rO- right occipital, rP- right parietal, rT-right temporal, rT-P- right tempo-parietal.

To calculate minimum sample size, we estimated mean ± standard deviation for the effect (cueing error) in the control group to be 0.1⁰±0.5⁰ based on results reported healthy participants in our previous study (Odoj and Balslev, 2016). With a ratio of k=5 between the sample sizes of neglect vs pooled control group and a type I error alpha=0.05, a one-tailed, independent samples t-test will detect a 1⁰ of difference in cueing error between controls and neglect patients in (1-β)=80% of cases for a neglect group of minimum N=3 patients (Chow et al., 2008; Rosner, 2010).

Participants consent to take part in the study was obtained according to the Declaration of Helsinki and the study was approved by the local Ethics Committees at the University of Tuebingen and the University of St Andrews.

### 2.2 Experiment 1. cross-modal attention

#### 2.2.1 Design

Participants discriminated a target (letter “A” or “H”) on a computer screen. The experimenter placed the participant's right, index finger just below the horizontal line where targets were presented. The finger was hidden from view and remained at the same position throughout a block of trials. Participants were instructed to direct attention to the location of the finger. We measured voice-reaction time for visual discrimination. To determine the locus of attention, we calculated the difference in reaction time for visual discrimination in the presence vs. the absence of the location cue. The procedure is described in more detail in (Odoj and Balslev, 2016). The locus of attention was the screen location where the cue caused the largest decrease in the reaction time. Cueing error was defined as the signed difference between the locus of attention and the actual location of the finger. If left neglect patients show a systematic, ipsilesional shift in the locus of attention relative to the cue, one would expect a cueing error in that direction. In order to be able to use reaction time alone as an index of performance, the analysis was restricted to locations in the right hemispace where neglect patients' accuracy was at ceiling, and not significantly different from the control groups.

#### 2.2.2 Setup

**Fig. 3:**
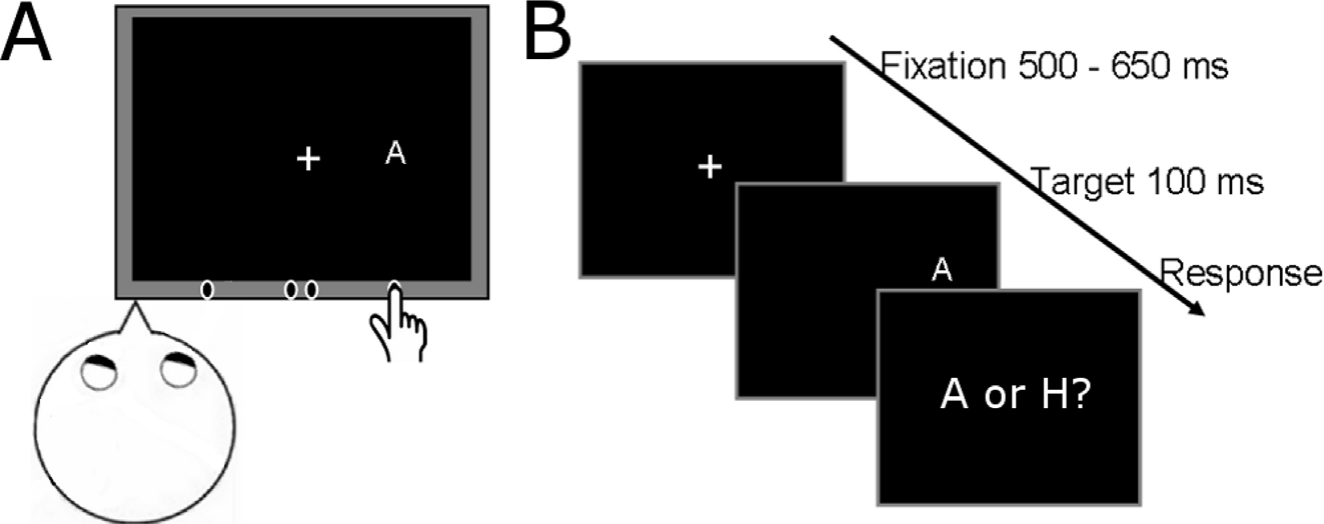
**Setup and task for the cross-modal attention experiment. A.** Participants fixated at +19° from the body/head midline. A hand proprioceptive cue (the participants' right index finger, hidden from view) was positioned at one out of four possible locations: 8°, 18°, 20° or 30° to the right from body-midline. **B.** A target letter, ‘A’ or ‘H’ was presented for 100 ms at one out of seven possible locations, at −3, −2, −1, 0, +1, +2 and +3 degrees horizontally from the finger. Participants named the letter as fast and accurately as possible.

Participants sat with their head fixed in a chin rest and cheek pads. A cathode ray tube (CRT) display was placed at 45 cm in front of them (Fig. 3A).

The CRT was centered 19° to the right from the body and head midline. A transparent sheet (Plexiglas) was mounted 5 cm in front of the CRT screen. Participants had their right index finger on a wooden ledge attached to the Plexiglas immediately under the location where the targets would appear. The finger was placed at one out of four possible cue positions, 8°, 18°, 20° or 30° to the right from body-midline and covered with black cloth. The experiment took place in total darkness, so the participants had no visual information about the location of their finger.

At the beginning of the trial, participants fixated on a central cross (white, 1°×1°) presented on a black background (Fig. 3B). Fixation was verified with a head-mounted EyeLinkII eye tracker in front of the right eye. After a random interval lasting between 500 and 650 ms the fixation cross disappeared. A hundred milliseconds later, a target letter (‘A’ or ‘H’, subtending 1°) appeared and stayed on the screen for another 100 ms. The target could appear at seven possible locations, at −3°, −2°, −1°, 0°, 1°, 2°, 3° from the cue. The target letter was presented with equal probability (eight times) at each of the seven possible target locations. Additionally, three trials showed target letters at random locations, further away from the cue. They were instructed to name the letter as fast and accurately as possible. Accuracy and voice reaction time were recorded.

Trials with the same cue location were grouped in blocks. Each block consisted of 59 trials (eight trials for each of the seven target positions + three random positions). Trial order was pseudo-randomized. At the end of each block, participants were instructed to close their eyes. Then the experimenter moved the participants' index finger at the next cue location and started a new block. The participants completed four cued blocks in the following order: cue at 30°, 8°, 18° and 20° from body midline.

To assess the baseline distribution of attention as well as visual accuracy, participants performed the same visual discrimination task in the absence of a cue. Participants’ right index finger rested in front of their body midline. Target letters were presented on the screen at all locations tested in the cued blocks. These locations were probed in random order, four times each. The uncued block consisted of 92 trials. This block was performed either before or after the cued blocks, randomized across participants.

The finger position cue in this experiment was not predictive for the location of the target, nor did it appear suddenly at that location. Although traditionally, these characteristics have been associated with an efficient cue (Driver and Spence, 1998; Posner et al., 1980), some studies show that they may not be absolute prerequisites. An arrow or a word (e.g.“left”) can direct participants' attention even in conditions when these cues do not predict the location of a subsequent target (Hommel et al., 2001; Pratt and Hommel, 2003; Tipples, 2008). Furthermore, one's own hand is a salient position cue (Reed et al., 2010, 2008, 2006). When the participant's own hand is hidden from view and placed in the vicinity of a computer screen, visual targets presented nearer the location of the hand are detected faster than targets presented further away (Reed et al., 2006). For these reasons we assumed that the participants' own finger will be an efficient attention cue in this task. This assumption was tested both here and in a previous study in which the same task was used (Odoj and Balslev, 2016). A signature of attention is the improvement in visual discrimination for retinally identical stimuli in the presence vs. the absence of the finger position cue. Inspection of reaction time data in the control groups showed a minimum at the exact location of the cue (Fig. 6).

#### 2.2.3 Statistical analysis

We calculated the difference in reaction time for visual discrimination in the presence versus the absence of the cue. The locus of attention was defined as the center of mass of all locations that showed a decrease in reaction time in the presence of the cue. The center of mass was calculated as the mean of these locations, after weighting each location with the magnitude of the cueing effect there. To separate out an eventual effect due to practice (i.e. an improvement of reaction time that was common to all target locations within a block), we pre-processed the data by subtracting the mean from each value therefore normalizing the scores within each block.

After checking the data for normality with Shapiro-Wilk test, mean cueing error for each cue location was compared between neglect patients and control groups using the appropriate test for two unrelated samples. Under the hypothesis that neglect patients’ loci of attention are displaced ipsilesionally relative to the cues, one would predict a rightward cueing error. One-tailed tests were used to test this prediction.

### 2.3 Experiment 2. pointing

#### 2.3.1 Design

Experiment 2 investigated whether the cueing error in Experiment 1 reflected a more general inability to locate the visual targets relative to the body, such as for instance a hand proprioceptive deficit. Participants pointed to visual targets presented at the exact same locations as the finger position cues in Experiment 1. Participants used their right, ipsilesional, index finger, the same finger that was used in Experiment 1 as a cue. No visual feedback was available. A general misalignment of hand proprioceptive and visual locations would cause errors in movement end-points.

#### 2.3.2 Setup

The setup was like in Experiment 1. Additionally, a position sensor (Polhemus Fastrak) was fixed on the tip of the participants’ right index finger.

Participants fixated at 19° from body midline, while a position marker was attached to their right index finger. A target (the letter ‘X’) was presented for 100 ms at one out of four possible locations, 8°, 18°, 20°, 30° from body midline. **B**. After target presentation, participants closed their eyes and pointed to the location where the target appeared. Finger location was recorded, and then the finger was passively moved back to the resting position.

**Fig. 4:**
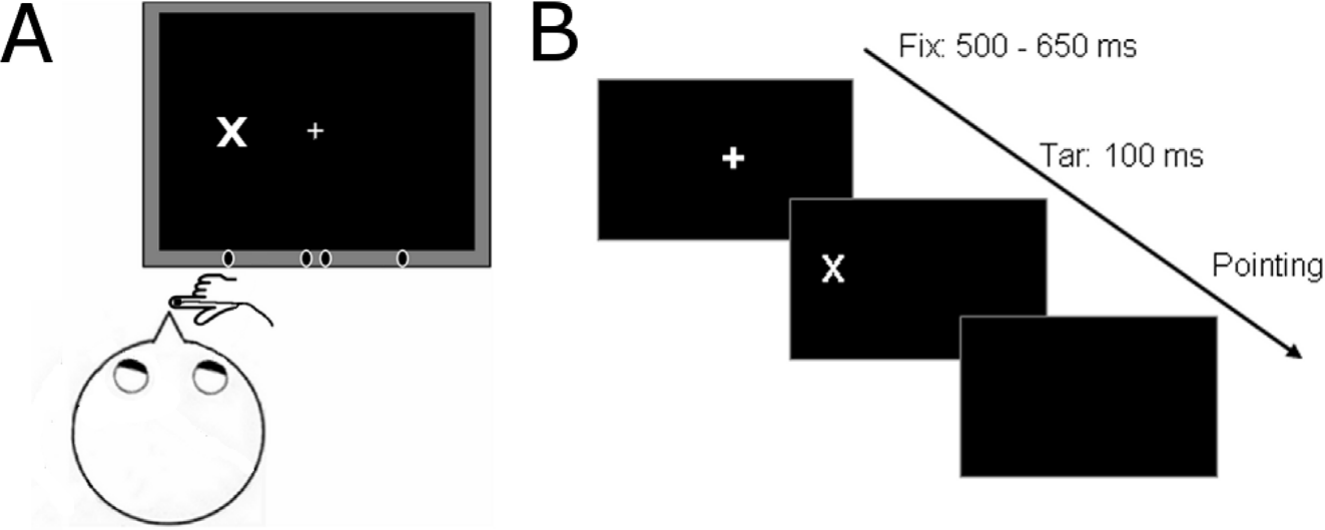
Setup and task for the visual open-loop pointing experiment. A.

At the beginning of the trial, the participants fixated on a central cross (1° × 1°, white on black background). Fixation was verified with the head-mounted eye tracker. Participants’ left index finger rested in front of their body midline (Fig. 4A). After 500–650 ms (randomized) the fixation cross disappeared. A hundred milliseconds later, a target letter (‘X’, subtending 1°) appeared and stayed on the screen for another 100 ms. The target could appear at 4 possible locations, at 8°, 18°, 20° or 30° from body midline. The participants were instructed to close their eyes and point as accurately as possible at the remembered location of the target. The reaching movement stopped on the wooden ledge, when the finger touched the Plexiglas. Participants were allowed to adjust the position of their finger until they felt the finger was pointing exactly towards the target. The instruction was given to encourage the use of hand proprioceptive feedback to adjust finger position until it matched the location of the visual target. The visual targets were briefly flashed, remembered and not present while pointing occurred and no visual feedback of hand movement was available. Finger position was then recorded and the experimenter moved the finger back to the resting position at body-midline.

Like in the cross-modal attention experiment, the trials in this control pointing experiment were blocked and the participants moved their finger to the same location throughout the block. The order of the blocks was the same across all participants (target at 30°, 8°, 18°, and 20° from body midline). Each block consisted of 6 trials.

Participants did not receive visual feedback of their pointing movement, and there was no learning across blocks in either group. Mean slope of the linear regression line was 0.02 in neglect, 0.04 in the patient control group and −0.39 in the healthy elderly controls. These values were not significantly different from zero in either group (one sample t-tests, all p's>0.3).

#### 2.3.3 Statistical analysis

Pointing error was calculated as the signed difference finger location minus target location in the horizontal plane at the end of the movement. After checking normality, mean pointing error for each target location was compared between neglect and control groups using appropriate independent-samples tests.

### 2.4 Eye tracking

The position of the right eye was recorded with a head mounted tracker (EYELINK II, SR Research Ltd., Ottawa, Canada) that sampled pupil location at 250 Hz. The tracker was calibrated using a 3 × 3 grid. Eye position time series were parsed into fixations, blinks, and saccades using the SR EyeLink detection algorithm and then analyzed off-line. The algorithm was set to detect saccades with amplitude of at least 0.5°, using an acceleration threshold of 9500°/sec^2^ and a velocity threshold of 30°/sec. Trials with a mean deviation of more than 1.5° visual angle from fixation within 50ms before target presentation were discarded.

### 2.5 Data availability

The data that support the findings of this study are available from the corresponding author, upon reasonable request*.*

## 3 Results

### 3.1 No significant difference in visual accuracy between neglect patients and controls in the right hemispace

**Fig. 5:**
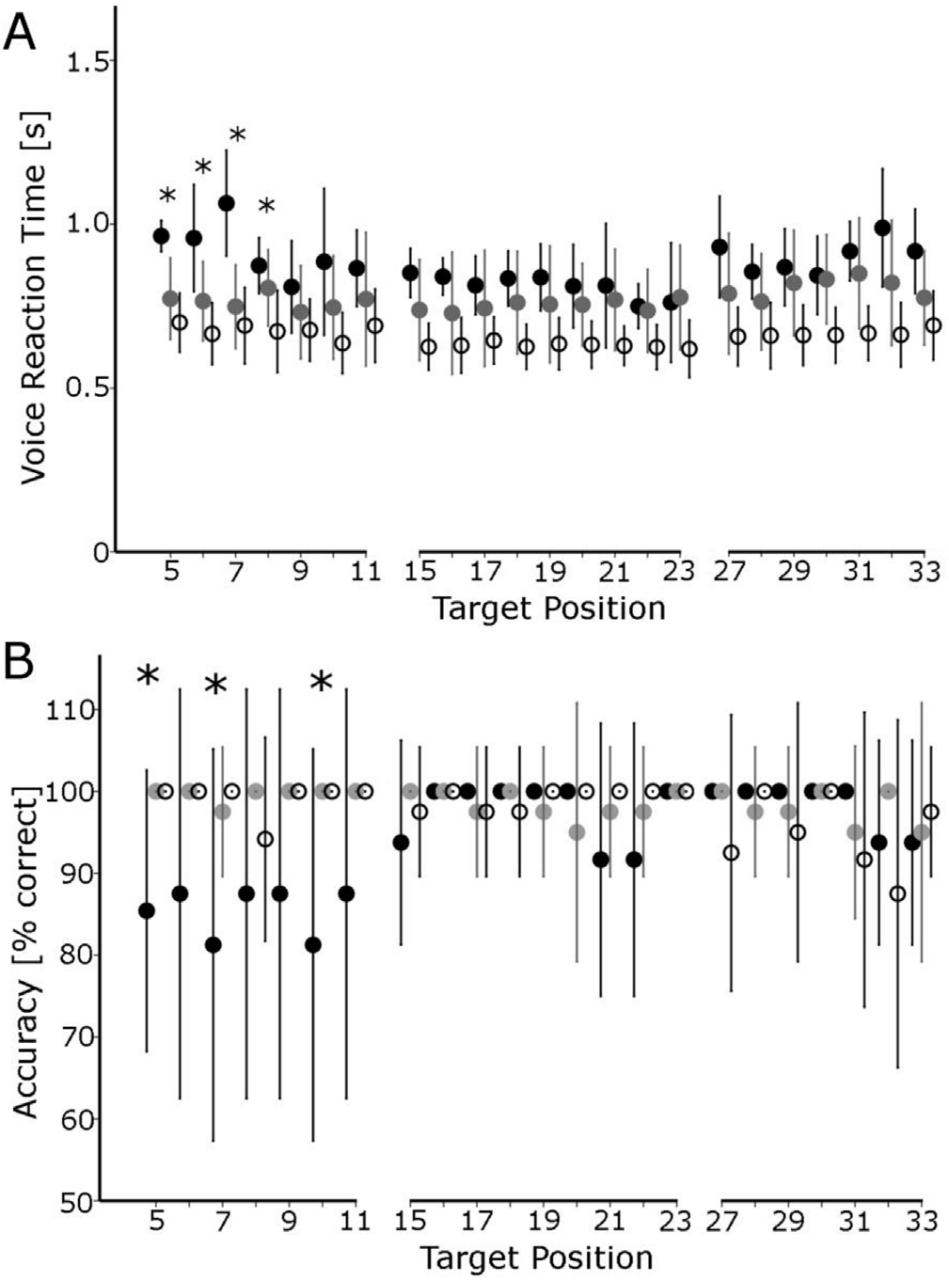
**Reaction time and accuracy for visual discrimination in the right body hemispace in the absence of a visual cue.** Neglect patients' (●) voice reaction time (A) and accuracy (B) was not significantly different than that of the patient control group () and healthy controls (◯) for targets located further than 11° to the right of the body midline (error bars show one standard deviation, * denotes p< 0.05, independent samples t-tests, neglect vs. control, for either control group).

As expected, neglect patients were significantly slower and less accurate than the healthy and the patient control group for the left-most targets. However, their performance for the targets situated further than 11° to the right of the body-midline was not significantly different from that of the control groups (Fig. 5). We found significant longer reaction times in neglect patients for the leftmost target locations, but not from 11° rightwards (independent samples t-tests, all p's< 0.042 for targets located from 5° to 9°, and all p's> 0.119 for target locations from 11°-33°). Likewise, visual accuracy was decreased for target locations at 5°, 7° and 10° (all p's< 0.047) whereas for all other locations visual accuracy was not significantly different from that of the control groups ( all p's> 0.116). The analysis of the cueing error was limited to target locations beyond 11° (cue at 18°, 20° and 30°). At these locations, the neglect patients' accuracy and reaction times were not significantly different from the control groups. With accuracy at or near ceiling for all groups (Fig. 5B), differences in reaction time alone are valid indicators of the difference in visual perception between groups.

**Fig. 6:**
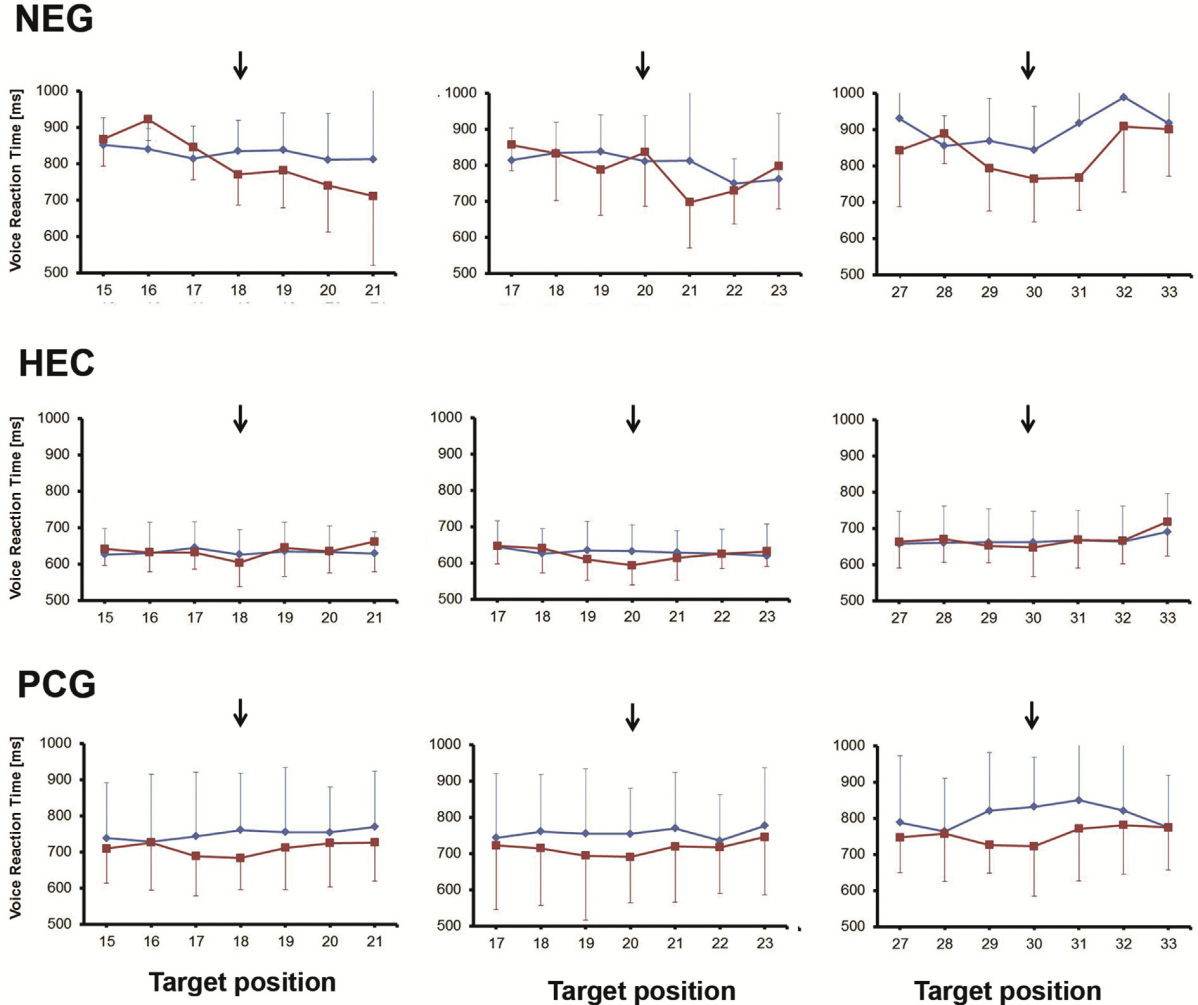
**Reaction time for visual discrimination in the cross-modal attention task in the presence** () **and the absence of the finger position cue** (). The arrow indicates the location of the cue in trials when the cue was present. NEG – patients with spatial neglect, HEC – healthy elderly controls, PCG – patient control group. Error bars show one standard deviation.

Inspection of the average reaction times (Fig. 6) showed a minimum in the voice reaction time at the exact location of the finger position in the two control groups for all tested locations. No such minimum was found in the condition without a finger position cue. This improvement in visual discrimination at the location of the finger in non-neglect participants confirms that finger position acted as an efficient spatial cue, directing attention to its location.

### 3.2 Rightward shift of loci of attention relative to the finger position cues in spatial neglect

In the control groups (healthy elderly controls – HEC and patient control group – PCG) the lowest value for the average voice reaction time was measured at the location of the finger position cue for all tested cue locations (18⁰, 20⁰ and 30⁰). In contrast, in the patients with spatial neglect, a minimum was usually observed for targets located to the right of the finger position cue (Fig. 6).

**Fig. 7:**
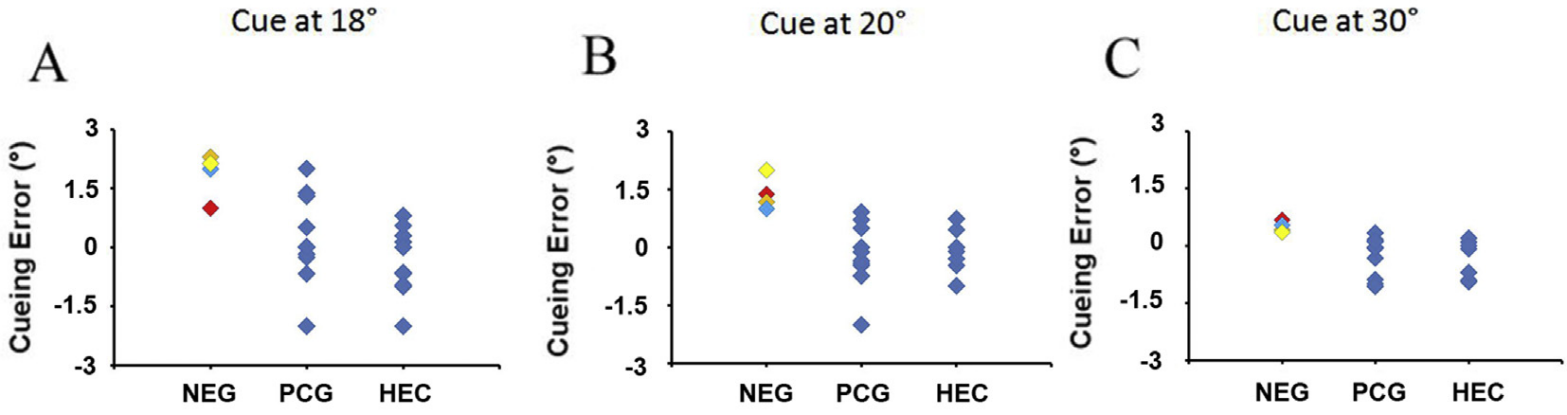
**Neglect patients show a rightward shift of the loci of attention in the visual space relative to the finger position cue.** The locus of attention was defined as the location with the largest advantage in reaction time for visual discrimination in the presence of the cue. The cueing error in neglect patients was in average 1.9°, 1.4° and 0.5° when the cue was presented at 18°, 20° and 30° from the body midline respectively. This was significantly different from the error in the control groups (p<0.001, one-tailed, independent samples t-tests and Mann-Whitney U test). NEG-neglect; individual patients with spatial neglect KS-, ASG-, SG-, UH-; PCG - patient control group; HEC – healthy elderly controls.

**Fig. 8:**
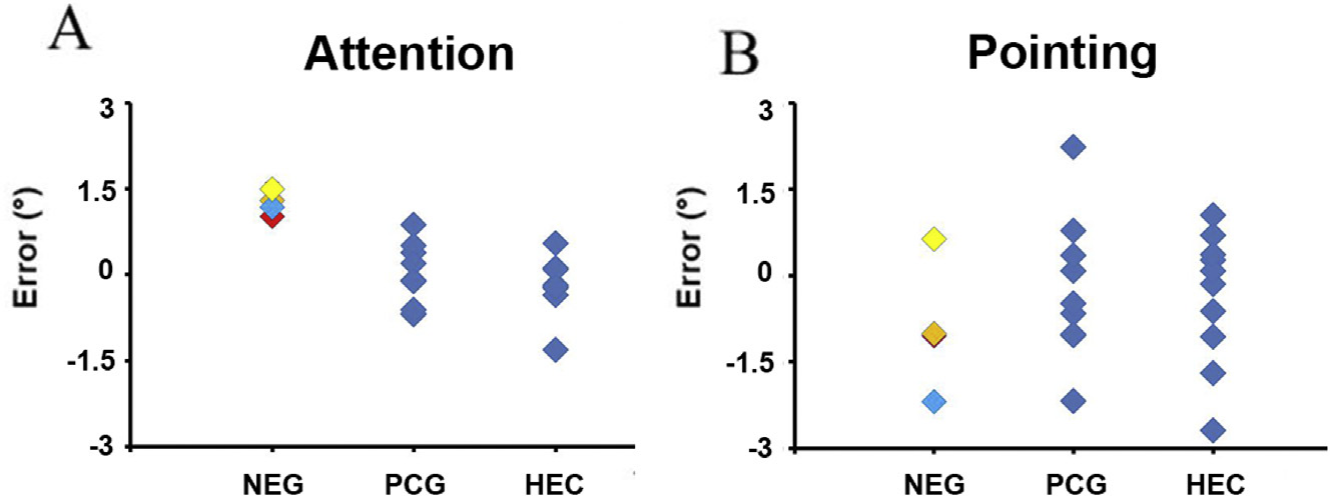
**Neglect patients show a rightward shift that is selective for the allocation of attention.** Their locus of attention in the visual space was in average at 1.25° right of the finger position across all tested locations (18°, 20° and 30° from the body midline). There was no statistically significant difference open-loop pointing error between neglect and control groups at any of these locations, which rules out a general error in coordinate transformations as an explanation of the results. NEG-neglect; individual patients with spatial neglect KS-, ASG-, SG-, UH-; PCG-patient control group; HEC – healthy elderly controls.

The comparison between neglect and either control group showed a larger, rightward cueing error in patients with spatial neglect (Fig. 7, Fig. 8). There was no statistically significant difference between the two control groups with respect to cueing error at either location (independent samples t-tests for cues at 18⁰ and 20⁰, two-tailed, both p's>0.2 and Mann-Whitney U test for cue at 30⁰ p>0.6). We compared therefore the cueing error for neglect patients with that of the pooled control group consisting of both healthy and patient controls. This comparison showed a significantly larger cueing error in the neglect group for each of the three cue locations (independent samples t-test for cue at 18⁰ and 20⁰, one-tailed, both p's < 0.001 and Mann-Whitney U test, p<0.001 for cue at 30⁰). Regression analysis showed a statistically significant linear decrease of cueing error from left to right, i.e. cueing error was largest when the cue was presented at 18° (beta coefficient = −0.11, t=−4.5, p=0.001).

### 3.3 The rightward cueing error occurred in each individual neglect patient

Single-case comparisons (Crawford and Howell, 1998) of the average cueing error across target locations (18°, 20° and 30° from the body midline) showed a significant rightward cueing error in each of the four neglect patients relative to the pooled control groups (one-tailed, p=0.02, 0.01, 0.007 and 0.003).

### 3.4 No difference in response accuracy across groups

In each of the three cued blocks, neglect patients were as accurate as the control groups. Mann Whitney U test showed no significant difference in accuracy across groups for any finger location and any pairwise comparison between the three groups (all p's > 0.18). Likewise, there was no significant difference between neglect vs. pooled control group (all p's>0.22).

### 3.5 No difference between the groups in the ability to fixate

Neglect patients had 13.87%± 7.2 of discarded trials with a break in fixation. These values were 12.2%± 5 for the patient control group and 9%± 6.8 for healthy controls. A one-way ANOVA showed no significant difference between groups (F (2, 21) = 1.184, p=.326).

### 3.6 No difference between groups in open-loop reaching error

One explanation for the rightward cueing error in neglect patients could be a general error in locating visual targets relative to the body. To investigate whether the findings were specific for spatial attention or more general for the coordinate transformation between visual and proprioceptive locations, we measured the error during pointing to a visual target in the absence of visual hand feedback. Pointing error was not significantly different from zero in any group (one sample t-tests, all p's>0.21). We found no statistically significant difference in pointing error between the neglect and control groups (Fig. 8). Because there was no statistical difference in mean pointing error between the two control groups (independent samples t-tests for target at 20⁰ and 30⁰, two-tailed, both p's>0.6 and Mann-Whitney U test for target at 18⁰ p>0.5), these two groups were pooled together. The comparison of the pointing error in neglect patients vs. pooled control group did not return any statistical significant difference for either target location (independent samples t-test for target at 20⁰ and 30⁰, one-tailed, both p's>0.17 and Mann-Whitney U test, p>0.15 for 18⁰). Likewise, single-case comparisons (Crawford and Howell, 1998) showed no statistically significant difference between any of the four neglect patients and the pooled control groups (one-tailed, p=0.28, 0.07, 0.29 and 0.21). These null results are in line with previous studies, showing accurate visual localization in neglect patients during reaching with no visual feedback (Himmelbach and Karnath, 2003; Rossit et al., 2009). Pairwise comparisons between mean cueing and pointing error showed a statistical significant difference for the neglect group only (one tailed, paired-samples test, p=0.011 for the neglect group, for the other two groups, both p's>0.4).

## 4 Discussion

The main finding of this study was a rightward displacement of the attention loci in the visual space relative to finger position in patients suffering from left spatial neglect. In these patients, the hand proprioceptive cues had the largest benefit for visual discrimination when the visual targets were located at 0.5°–1.9° to the right of the cues. This ipsilesional, rightwards, displacement of the attention loci was consistently observed for all three tested cue locations and in all four neglect patients. In contrast, the controls showed an advantage in visual discrimination at the exact location of the finger cue. This consistent, ipsilesional shift in spatial neglect was selective for the allocation of attention. The patients’ error in open loop reaching with their right index finger to the remembered location of the visual targets was not significantly different from the control groups, suggesting a normal ability for visual localization for reaching movements.

These results suggest a new, additional mechanism for the attention imbalance in spatial neglect, an error in the coordinate transformation for the attentional priority map. If the rightward displacement observed here occurs throughout the space, an ipsilesional displacement of all attention loci relative to sensory stimuli that demand attention would lead to a right-left imbalance in the allocation of attention.

### 4.1 Neural mechanisms underlying the ipsilesional shift in attention loci

The right shift of the attention locus in the visual space relative to the finger position suggests that the eccentricity of the fixation cross relative to the body midline is overestimated. This is indicative of a right-shift in perceived gaze direction for the attentional priority map.

Our previous work shows a causal connection, from a distortion of the oculoproprioceptive signal in the somatosensory cortex after rTMS, to a systematic displacement of attention loci in the visual space in response to a hand proprioceptive cue (Odoj and Balslev, 2016). Because previous studies including ours have found that the movement of a hidden hand to visual targets remains accurate in the presence of an oculoproprioceptive distortion (Lewis et al., 1998; Odoj and Balslev, 2016), we proposed that spatial representations for reaching and attention rely on different combinations of gaze direction signals (Odoj and Balslev, 2016). It is important to note however, that the current results cannot be explained by a mere distortion of the oculoproprioceptive signal from the somatosensory cortex. After a somatosensory cortex lesion the angle of gaze is underestimated due to a reduced oculoproprioceptive input. This would cause a shift in the perceived direction of gaze direction towards the body midline (Balslev et al., 2013; Odoj and Balslev, 2016). Such a shift is in the opposite direction to that we observed here.

A distortion of the gaze direction input to the attentional priority map in spatial neglect could result from a dysfunction in any of the signals that convey the rotation of the eyes in the orbits or the head on the trunk: proprioception from eye or neck muscles, efference copy (corollary discharge) for these muscles or vestibular signals. The displacement did not affect the patients’ ability to locate visual targets relative to the body when the index finger acted as an effector for reaching rather than a cue for attention. This control experiment rules out an alternative explanation of the current findings. A general error in the coordinate transformation from hand proprioceptive to visual space, such as for instance that following a distortion in hand proprioception would cause similar errors in both tasks. We suggest therefore that the lesion in spatial neglect affects selectively the gaze direction estimate fed into the attentional priority map. The neural pathways for the gaze direction signals (vestibular, proprioceptive, visual and corollary discharge) to the attentional priority map and the impact of a lesion along these pathways on the signals that convey gaze direction are not well understood.

Electrophysiological recordings in the monkey show that neurons in the parietal lobe's area LIP that respond to task relevant stimuli and not to distractors, are sensitive to both the retinal location of these stimuli and to the direction of gaze (Andersen and Mountcastle, 1983; Morris et al., 2013). These neurons are thought to implement the coordinate transformations between retinal and body-centered locations. The source of the gaze direction signal to the gain-field neurons is currently unknown. Single cell recordings show that this gaze direction signal updates slowly, at least 150 ms after a saccade (Xu et al., 2012). The delay would be compatible with a proprioceptive or vestibular source. During the delay in which the gain field signal is unreliable, the monkeys' performance in a double step saccade task seems unaffected. The double step saccade task requires an extraretinal signal of eye position to compute the retinal location of the second saccade target. This discrepancy between neural activity of the gain field neurons and behaviour suggests that the necessary computations for the double step saccade task are supported by different neurons that use a faster gaze direction signal, presumably corollary discharge (Xu et al., 2012). One could speculate that while neural computations necessary for fast behaviour, such as double-step saccades or hand movements, relies on the predictive corollary discharge, gain field neurons use a delayed, but more accurate vestibular/proprioceptive gaze direction signal to implement slower behaviour, such as the allocation of attention around the body. The lesion in spatial neglect could distort the gaze direction input to these neurons.

### 4.2 A new hypothesis for the mechanism of the attention imbalance in spatial neglect

The idea that an error in coordinate transformation between retinotopic and the body-centred space might underlie some of the symptoms in spatial neglect is not new (Jeannerod and Biguer, 1987; Karnath, 1997; Vallar, 1997). Perceived rotation of the egocenter in patients with spatial neglect (Karnath, 1997) could cause an ipsilesional shift in the baseline allocation of attention. A steady-state shift in spatial attention however, would affect equally both conditions of Experiment 1, with and without the finger position cue. The locus of attention here was defined by the difference in reaction time between these two conditions. Because a change in baseline attention would be common to both conditions, it cannot impact on the reaction time difference. We argue therefore that the shift of attention loci observed here reflects a systematic mislocalization of the peaks on the attentional priority map relative to attention cues, rather than a change in baseline attention. While our results are in line with the idea of a faulty coordinate transformation in spatial neglect, they also suggest a new mechanism for the attention imbalance. The attentional priority map misrepresents the location of the cues by displacing their corresponding attention loci towards the ipsilesional space.

Likewise, others have suggested that patients with spatial neglect fail to retain cue locations accurately (Pierce and Saj, 2018; Pisella and Mattingley, 2004). Our hypothesis is broadly in line with this suggestion. However, a remapping failure across eye movements cannot be responsible for current findings because the distortion of the attentional priority map was observed at fixation, in the absence of eye movements. Previous studies in spatial neglect that report an inability to recall visual locations across saccades have used visual localisation tasks such as target directed saccades (Duhamel et al., 1992; Heide and Kömpf, 1998; Husain et al., 2001). Because different spatial representations use different combinations of eye position signals (Odoj and Balslev, 2016), visual localisation tasks may not be an accurate indicator of the attentional priority map. To the best of our knowledge, this is the first time a study has measured directly where patients with spatial neglect deploy attention in response to a cue. While their visual localisation in the absence of eye movements was normal, the patients misrepresented cue location showing perceptual enhancement at irrelevant locations, positioned further towards the ipsilesional space than the finger position cue.

Finally, the finding of a systematic displacement of attention loci in the right, ipsilesional hemispace of the patients with left neglect cannot be explained by a reduced, but otherwise accurate attentional priority map (Pouget and Driver, 2000).

By showing distorted gaze input to the attentional priority map we confirm a role of gaze direction signals in the pathophysiology of spatial neglect. We suggest that the ipsilesional displacement of attention loci relative to attention cues is a mechanism for the attention imbalance.

### 4.3 Sample size

Stringent requirements for gaze fixation and accuracy in this study limited the number of stroke patients who could complete the experiment. We report data from all neglect patients who were able to carry out the tasks (N=4). Their performance was compared with that of ten age-matched controls and ten stroke patients without spatial neglect. The study was meticulously designed using a task and an analysis method previously tested in healthy participants (Odoj and Balslev, 2016) and had >80% power to detect 1⁰ of difference in cueing error between the neglect patients and the pooled control group (Chow et al., 2008; Rosner, 2010). Furthermore, because of the small number of neglect patients who could perform to criteria, we also carried out single subject analyses that contrasted performance in each individual patient with that of the pooled control group, using statistical methods specifically designed for this purpose (Crawford and Howell, 1998). These analyses confirmed the results of the group analysis.

The neglect group consists of one chronic patient (>5 years post stroke, ASG) and three subacute patients (<1 month post stroke, KS, SG and UH). In contrast, the control patient group is all chronic stage (4–17 months post stroke). Inspection of our chronic neglect patient ASG's individual data show a numerical value for the error in both attention and pointing tasks that was within the range defined by the performance of the other patients with spatial neglect (Fig. 7BC and 8), or even larger (Fig. 7A). The data in this small sample of neglect patients therefore does not suggest that the difference in performance between neglect and patient control group reflects the difference in the time after stroke.

### 4.4 Effect size

The angular displacement observed here was ∼1° in average. Regression analysis showed a significant, linear increase in cueing error from right to left, with the leftmost cue being displaced by 2° and the rightmost cue by 0.5° relative to the finger position cue. In this study only locations in the patients’ right hemispace, where their accuracy for visual discrimination was at ceiling, could be assessed. The leftmost cue was at 18° to the right of the body midline. Under the assumption that the cueing error would follow the same trend towards the left hemispace, one would predict a larger effect in the left hemispace. In line with this conjecture, recent experiment has found that distractors presented in the left, neglected hemifield paradoxically facilitate detection of a rightward target (Vossel and Fink, 2016). The authors suggest their finding is likely to reflect a transformation of the contralesional stimulation into a saliency signal contributing to facilitated information processing in ipsilesional space.

In summary, we report that patients with spatial neglect have a rightward shift of the attention loci in the visual space relative to hand proprioceptive cues. This indicates an error in the gaze direction input that transforms somatosensory to visual locations. The error was specific for the attentional priority map, because reaching to visual targets with the hidden hand was relatively unaffected. A systematic, ipsilesional displacement of attention loci could cause a left-right attention imbalance.

## Funding

This work was supported by the Danish Medical Research Councils [grant number 09–072209] and an Institutional Strategic Support Fund at the University of St Andrews from the Wellcome Trust. The funding sources had no involvement in study design; in data collection, analysis and interpretation; in writing of the report and in the decision to submit the article for publication.

## Conflicts of interest

The authors declare no competing financial interests.

## CRediT authorship contribution statement

**Daniela Balslev:** Conceptualization, Data curation, Formal analysis, Funding acquisition, Methodology, Project administration, Resources, Software, Supervision, Validation, Visualization, Writing - original draft, Writing - review & editing. **Bartholomäus Odoj:** Conceptualization, Data curation, Formal analysis, Methodology, Resources, Software, Validation, Visualization, Writing - original draft, Writing - review & editing.
